# Human induced pluripotent stem cells and male infertility: an overview of current progress and perspectives

**DOI:** 10.1093/humrep/dex369

**Published:** 2018-01-05

**Authors:** Fang Fang, Zili Li, Qian Zhao, Honggang Li, Chengliang Xiong

**Affiliations:** 1 Family Planning Research Institute, Tongji Medical College, Huazhong University of Science and Technology, 13 Hangkong Road, Wuhan 430030, China; 2 Center for Reproductive Medicine, Wuhan Tongji Reproductive Medicine Hospital, 128 Sanyang Road, Wuhan 430013, China

**Keywords:** induced pluripotent stem cells, embryonic stem cells, male infertility, primordial germ cells, germ cell differentiation, reproductive medicine, gene editing, extracellular vesicles

## Abstract

Recently, significant progress has been made in ART for the treatment of male infertility. However, current ART has failed to help infertile patients with non-obstructive azoospermia, unless donor sperm is used. In fact, most couples wish to have their own genetically related child. Human induced pluripotent stem cells (hiPSCs) can be generated from patients’ somatic cells and *in vitro* derivation of functional germ cells from patient-specific iPSCs may provide new therapeutic strategies for infertile couples. The overall developmental dynamics of human primordial germ cells are similar to that in mice, but accumulating evidence suggests that there are crucial differences between human and mouse PGC specification. Unlike mouse iPSCs (miPSCs) in naive state, hiPSCs exhibit a primed pluripotency which possess less potential for the germ cell fate. Based on research in mice, male germ cells at different stages have been derived from hiPSCs with different protocols, including spontaneous differentiation, overexpression of germ cell regulators, addition of cytokines, co-culture with gonadal cells *in vitro* and xeno-transplantation. The aim of this review is to summarize the current advances in derivation of male germ cells from hiPSCs and raise the perspectives of hiPSCs in medical application for male infertility, as well as in basic research for male germ cell development.

## Introduction

Infertility is a global public health concern with a high prevalence in couples at reproductive age (Inhorn and Patrizio, 2015). Male infertility accounts for approximately half of all cases of infertility, and non-obstructive azoospermia is the most severe form (Miyamoto *et al.*, 2015). Besides genetic factors, azoospermia also occurs due to injuries, exposure to toxicants, immune-suppressive and anticancer treatments (Skakkebaek *et al.*, 2001; Bhartiya *et al.*, 2014). However, a large proportion of infertile males are diagnosed as idiopathic with unknown causes, reflecting poor understanding of the mechanisms regulating spermatogenesis and sperm function in humans (Ferlin *et al.*, 2007).

Recently, significant progress has been made in ART for the treatment of infertility. However, current ART has been unable to help the infertile couples who lack functional gametes, unless donor gametes were used. In fact, most couples wish to have their own genetically related child (Ishii, 2014). With the rapid development of stem cell technology, the possibility to derive artificial gametes from human pluripotent stem cells may provide new therapeutic strategies for infertile couples.

Embryonic stem cells (ESCs) can differentiate into male germ-like cells *in vitro*, but they are genetically unrelated to the patients, and the sources of human ESCs (hESCs) are limited and accompanied by ethical issues about destruction of embryos (Devolder, 2010; Hou *et al.*, 2014). The ectopic expression of four transcription factors (OCT4, SOX2, KLF4 and MYC) leads to the reprogramming of somatic cells to induced pluripotent stem cells (iPSCs) which resemble ESCs in morphology, pluripotency marker expression and differentiation ability (Park *et al.*, 2008; Takahashi and Yamanaka, 2006). To some extent, human iPSCs (hiPSCs) are superior to hESCs for reproductive medicine application because there are few ethical issues and the sources are abundant. Furthermore, hiPSCs can be generated from patients’ somatic cells and are immuno-compatible for auto-transplantation. So the generation of patient-specific spermatozoa from hiPSCs will provide the foundation for future treatment of male infertility. However, hiPSCs may not faithfully recapitulate the characteristics of hESCs at both genetic and epigenetic levels (Bock, *et al.*, 2011; Doi, *et al.*, 2009). Especially, hiPSCs are reported to keep some epigenetic marks of the donor cell type from which they were reprogrammed (Kim, *et al.*, 2011).

All in all, the discovery of hiPSCs may not only lead to clinical approaches addressing infertility resulting from defects in gametogenesis but also provide an opportunity to investigate the molecular mechanism of human germ cell development. In this review, we summarize the current advances in derivation of male germ cells from hiPSCs and raise perspectives of hiPSCs in medical application for the treatment of male infertility, as well as for basic research into the mechanisms of human germ cell development.

## Discussion

### Specification of human male germ line cells

Primordial germ cells (PGCs) are founder cells of the germ line and are specified during early embryonic development in mammals. Mouse PGC (mPGC) specification has been studied extensively, which provides a valuable model for mammalian development. Briefly, mPGC specification is initiated by bone morphogenetic protein (BMP) and WNT signals from extra-embryonic tissues which induce the expression of PGC fate regulator genes in a few germ line competent cells of the early post-implantation embryo (Saitou *et al.*, 2005; Weber *et al.*, 2010; Yamaji *et al.*, 2008). The mPGCs at the base of the allantois begin to migrate and colonize the genital ridge, accompanied with genome-wide epigenetic reprogramming to erase imprints and other somatic epigenetic memories (Lawson and Hage, 1994; Tang *et al.*, 2016). Post-migration PGCs start sex-specific development and the male germ cells undergo mitotic arrest, indicating the end of the PGC stage of germ line development (McLaren, 2003).

The overall developmental dynamics of human PGCs (hPGCs) are similar to that in mice, but crucial differences exist. Human PGCs express several lineage regulatory genes that are absent in mPGCs, such as trophectoderm regulator TEA domain transcription factor 4 (TEAD4) and endoderm regulator SRY-box 17 (SOX17); also hPGCs lack the core pluripotency gene SOX2 but express naive pluripotency factors transcription factor CP2 like 1 (TFCP2L1) and Kruppel like factor 4 (KLF4) (de Jong *et al.*, 2008; Perrett *et al.*, 2008; Tang *et al.*, 2015). Intriguingly, SOX17 is a critical specifier of hPGC fate but is dispensable for mPGC specification (Hara *et al.*, 2009; Irie *et al.*, 2015). WNT signals induce the expression of eomesodermin (EOMES) to activate SOX17 for human PGC-like cells (PGCLCs) specification (Kojima, *et al.*, 2017). Knockdown of SOX17 induces the repression of PR/SET domain 1 (PRDM1) expression, indicating that PRDM1 acts downstream of SOX17 (Irie *et al.*, 2015). Additionally, it is known that a tripartite transcription factor network of PRDM1, PRDM14, and transcription factor AP-2 gamma (TFAP2C) represses the somatic fate and promotes the PGC specification in mice (Magnusdottir *et al.*, 2013). However, studies suggest that PRDM14 has a less prominent role in hPGC development, and more research is needed to verify the role of PRDM14 in human germ line development (Guo *et al.*, 2015; Sugawa *et al.*, 2015). Thus, SOX17 and PRDM1 contribute to the human germ cell development, whereas the role of TFAP2C and PRDM14 in hPGC specification remains to be fully addressed.

After colonizing the developing gonad in the genital ridge, hPGCs are known as gonocytes (Stukenborg *et al.*, 2010). Through crosstalk with surrounding somatic cells, gonocytes enter the male germ cell development path and become spermatogonia postnatally; thereafter, spermatogonia undergo mitotic proliferation until puberty, when meiosis is initiated to form final spermatozoa (Manku and Culty, 2015; Stukenborg *et al.*, 2014).

### Different pluripotency state between human and mouse iPSCs

Recently, two developmentally and functionally distinct types of pluripotency have been defined: the naive state and the primed state (Nichols and Smith, 2009; Petkova *et al.*, 2014). First, cells in the naive state are competent to form blastocyst chimeras; the presence of two active X chromosomes is an epigenetic signature of naive pluripotecy; naive cells express KLF2 and KLF4 in addition to core pluripotency factors, and naive markers like reduced expression 1 (REX1, officially known as ZFP42), nuclear receptor subfamily 0 group B member 1 (NR0B1) and fibroblast growth factor 4 (FGF4); the two types of pluripotent cells also respond differently to signal molecules, such as leukemia inhibitory factor/signal transducer and activator of transcription 3 (LIF/STAT3) and fibroblast growth factor/extracellular regulated kinase (FGF/ERK); more specifically, the differentiation potential of primed pluripotent cells into PGCs and mature germ cells is drastically different from that of naive pluripotent cells (Arabadjiev *et al.*, 2012; De Los Angeles *et al.*, 2012; Nichols and Smith, 2009, 2011).

Rodent ESCs established from pre-implantation blastocyst could be in a naive state, and rodent ESCs procured from the post-implantation epiblast could be in a primed state preparing for differentiation; however, hESCs only had one primed state (Najm *et al.*, 2011; Nichols and Smith, 2011; Tesar *et al.*, 2007). Mouse iPSCs (miPSCs) present a naive state of pluripotency similar to mouse ESCs (mESCs) derived from the inner cell mass, whereas hiPSCs are considered to show a primed state of pluripotency that resembles the post-implantation epiblast (Nichols and Smith, 2009; Theunissen *et al.*, 2016, 2014). In light of the disparate pluripotency states in miPSCs and hiPSCs, it may be misleading to translate the results achieved in rodent models to human research directly. However, Gafni *et al*. (2013) established the four-inhibitor-containing culture medium (4i medium) that could facilitate the derivation of naive hiPSCs.

### Derivation of male germ line cells from hiPSCs

Murine studies have provided substantial insight into the development of male germ cells from iPSCs both *in vitro* and *in vivo* (Cai *et al.*, 2013; Imamura *et al.*, 2010; Li *et al.*, 2014b; Yang *et al.*, 2012; Zhu *et al.*, 2012). Hayashi *et al*. (2011) made the remarkable finding that PGCLCs could be obtained from mESCs and miPSCs. The PGCLCs could be differentiated into spermatozoa *in vivo*, resulting in the birth of healthy offspring via intracytoplasmic sperm injection. Zhou *et al*. (2016) reported the generation of haploid male gametes from mESCs that could produce viable and fertile offspring. Notably PGCLCs derived from different mouse iPS cell lines exhibited different efficiency for spermatogenesis *in vivo* and some of the offspring died prematurely (Hayashi *et al.*, 2011).

In spite of progress in mice, differentiation of hiPSCs to male germ cells still presents a significant challenge. Unlike miPSCs in naive state, hiPSCs exhibit a primed pluripotency with less potential for the germ cell fate (Hayashi and Surani, 2009; Nichols and Smith, 2009). Therefore, it may not be surprising that the success rate of germ cell derivation from hiPSCs is much lower than that from miPSCs.

Based on research in mice, male germ cell induction from hiPSCs has been attempted with different protocols, including spontaneous differentiation, overexpression of germ cell regulators, addition of cytokines, co-culture with gonadal cells *in vitro* and xeno-transplantation (Table I). Park *et al*. (2009) reported the first successful attempt to create PGCLCs from hiPSCs by co-culturing with human fetal gonadal stromal cells and showed that the erasure of the genetic imprint did not initiate efficiently in PGCLCs. BMP signaling is demonstrated to be conserved for both human and mouse germ cell induction, and the addition of BMPs could induce germ cell differentiation from hiPSCs. By combining BMP addition with overexpression of members of the deleted in azoospermia (DAZ) gene family, hiPSCs formed meiotic cells with extensive synaptonemal complexes and post-meiotic haploid cells with a similar pattern of acrosin staining as observed in human spermatids (Panula *et al.*, 2011). Furthermore, Medrano *et al.* found intrinsic germ cell translational, rather than transcriptional factors could drive germ line formation from hiPSCs *in vitro*. With overexpression of VASA (officially known as DDX4) and/or deleted in azoospermia like (DAZL) (RNA-binding proteins), hiPSCs differentiated to PGCLCs and progression through meiosis was enhanced. They also found that ectopic expression of VASA resulted in recapitulation of some aspects of germ line reprogramming at the H19 locus (Medrano *et al.*, 2012). Unlike aforementioned studies with genetic manipulation, Eguizabal et al. (2011) and Easley *et al*. (2012) demonstrated that post-meiotic haploid cells could also be obtained from hiPSCs without the overexpression of germ line specific factors. Eguizabal *et al.* achieved complete differentiation of hiPSCs derived from different origins (keratinocytes and cord blood) and both genetic sexes into post-meiotic cells *in vitro* using a 3-step differentiation protocol. However, there was an imprinting re-establishment that was not complete in the differentiated cells. Easley *et al.* showed that hiPSCs could differentiate directly into post-meiotic, spermatid-like cells under standardized mouse spermatogonial stem cell (SSC) culture conditions. The haploid cells presented similar DNA methylation patterns to human sperm both on paternally and maternally imprinted genes (imprinted maternally expressed transcript (non-protein coding) (H19) and insulin like growth factor 2 (IGF2)).

**Table I:** The *in vitro* differentiation potential of human iPSCs into male germ cells.

Authors	Donor cells	Methods		Reporters	Isolation strategy	RNA markers	Protein markers	Results	Genetic and epigenetic analysis
Park *et al.* (2009)	Dermal fibroblasts	Co-culture with human fetal gonadal cells			SSEA1^+^/cKIT^+^/VASA^+^ and PLAP^+^/SSEA1^+^/VASA^+^	VASA, PRDM1, DPPA3, and DAZL	cKIT and VASA	PGCLCs	Incomplete imprint erasure
Panula *et al.* (2011)	Fetal- and adult-derived fibroblasts	BMP-induced culture and overexpression of the DAZ gene family		VASA:GFP reporter	VASA:GFP^+^	VASA, IFITM1, PELOTA, PRDM1A, GCNF, STELLAR, and DMC1	VASA, DAZL, SCP3, CENP-A and Acrosin	Meiotic cells and haploid cell	DNA content analysis, and FISH
Eguizabal *et al*. (2011)	Keratinocytes and cord blood	3-step methods (RA, FRSK, LIF, R115866)			CD9^+^/CD49f^++^/CD90^−^/SSEA-4^−^	VASA and Stra8	VASA, SCP3, γ-H2AX and Acrosin	Haploid gamete-like cells	DNA content analysis, FISH, and incomplete imprinting re-establishment
Easley *et al.* (2012)	Foreskin fibroblast	Standardized mouse SSC culture conditions			Isolation for haploid cells	VASA, DAZL, CXCR4, PIWIL1, and PLZF	VASA, DAZL, UTF1, CDH1, RET, GFRα1, PIWIL1, HIWI, SCP3, TP1, protamine 1 and Acrosin	Haploid spermatogenic cells	DNA content analysis, FISH, and similar parent imprints
Medrano *et al.* (2012)	Fetal- and adult-derived fibroblasts	Overexpression of VASA and/or DAZL and spontaneous differentiation		VASA:GFP reporter	VASA:GFP^+^	VASA, IFITM1, DAZL, PRDM1A, GCNF, GDF3, cKIT, PELOTA, SCP3, MLH1, DMC1, GDF9, and ZP4	VASA, CENP-A, SCP3 and Acrosin	Meiotic cells	DNA content analysis, FISH, and recapitulation of epigenetic reprogramming at the H19 locus
Durruthy-Durruthy *et al.* (2014)	Dermal fibroblasts	Ectopic expression of VASA	BMP4 treatment *in vivo*			NANOS3, VASA, and DPPA3	VASA, DAZ, DAZL, DPPA3, UTF1 and GFRα1	PGCLCs, and pre-meiotic germ cells	Epigenetic transition from 5-mc to 5-hmc
			Xeno- transplantation						
Ramathal *et al.* (2014)	Dermal fibroblasts from azoospermic and fertile men	BMP4, BMP8, RA, LIF *in vitro* Xeno-transplantation		VASA:GFP reporter	VASA:GFP^+^	VASA, PRDM1, PRDM14, DAZL, STELLA, IFITM3, and NANOS3	VASA, DAZL, STELLA, PLZF, UTF1 and DAZ	PGCLCs, and gonocyte-like cells	Global DNA demethylation
Irie *et al.* (2015)	Somatic cells from a fragile X male patient and normal female	BMP2 or BMP4, LIF, SCF, EGF, and ROCK inhibitor		NANOS3- mCherry reporter	NANOS3^+^/TNAP^+^	NANOS3, BLIMP1, TFAP2C, SOX17, STELLA, OCT4, and PRDM14		PGCLCs	
Sugawa *et al.* (2015)		BMP4, ActA, bFGF, LIF			TRA-1–81^+^/cKIT^+^	BLIMP1, STELLA, cKIT, STELLA, NANOS3, and TEX13B	BLIMP1 and STELLA	PGCLCs	Global progress of epigenetic reprogramming
Sasaki *et al.* (2015)	Dermal fibroblasts and PMBCs	Activin A, CHIR99021, BMP4, SCF, EGF, LIF		BLIMP1-2 A -tdTomato and TFAP2C-2 A -EGFP reporters	BLIMP1^+^/TFAP2C^+^ and EpCAM^+^/INTEGRINα6^+^	BLIMP1, TFAP2C, NANOS3, DPPA3, DDX4, and DAZL	BLIMP1, TFAP2C and SOX17	PGCLCs	Avoiding of somatic program and epigenetic reprogramming

**Figure 1:**
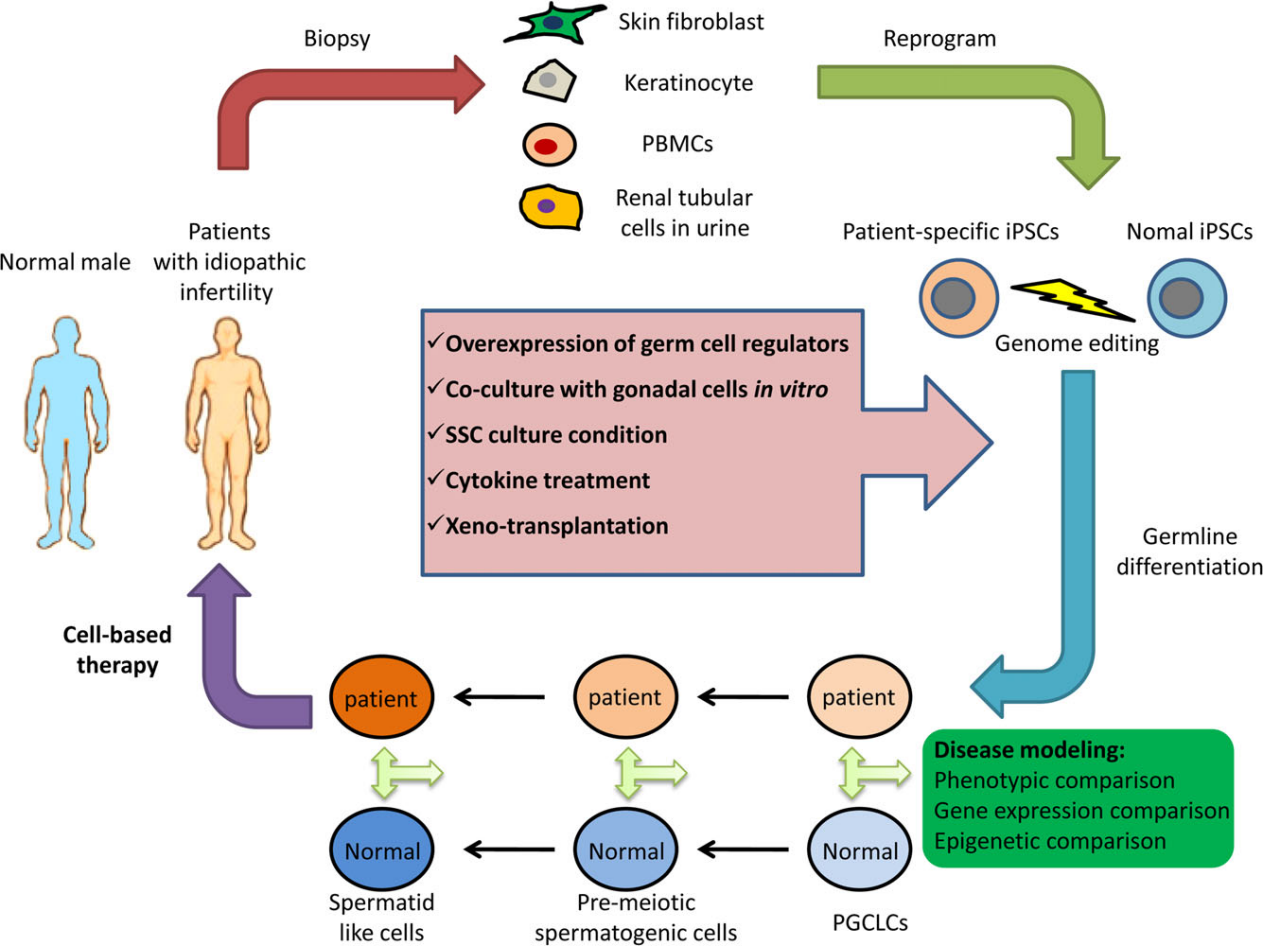
Derivation and application of patient-specific induced pluripotent stem cells (iPSCs) in male infertility. Different types of somatic cells derived from patients with idiopathic infertility are reprogrammed into iPSCs and then differentiated into male germ cells by multiple methods. If necessary, iPSCs with known genetic defects may be corrected by genome editing technology. These cells can be used for *in vitro* disease modeling, regeneration research and cell-based therapy. In disease modeling, comparison between patients- and normal derived cells potentially provides novel clues to the underlying mechanisms for idiopathic male infertility, which may further lead to the development of therapeutic strategies. PBMCs, peripheral blood mononuclear cells; SSC, spermatogonial stem cell; PGCLCs, human primordial germ cells-like cells.

It is important to point out that the gonadal environment *in vivo* is required for definitive and successful meiosis. However, transplantation of iPSCs or iPSC-derived cells into human testis is limited by ethical and safety issues. Thus, another significant method for male germ cell differentiation is xeno-transplantation of iPSCs into murine or even primate testis to evaluate their differentiation potential for germ line cells. In order to make use of the gonadal niche to promote human germ line formation *in vivo*, Durruthy-Durruthy *et al.* transplanted hiPSCs directly into the seminiferous tubules of germ cell-depleted immunodeficient mice. The transplanted iPSCs migrated to the basement membrane of the seminiferous tubule and 8 weeks after transplantation, the differentiated cells expressed PGC and pre-meiotic germ cell markers (Durruthy-Durruthy *et al.*, 2014). Interestingly, they found that iPSCs produced with different factors (addition of VASA to OSKM (OCT4, SOX2, KLF4 and MYC) or OSKM only) revealed divergent fates after xeno-transplantation. In contrast to OSKM cells, OSKMV-reprogrammed iPSCs showed greater germ cell forming potential and did not form tumors, while OSKM cells remained outside the seminiferous tubule proliferated extensively and formed tumors. Using the same method, the authors also transplanted iPSCs derived from azoospermic and fertile men to murine seminiferous tubules. Human iPSCs with azoospermia factor deletions produced significantly fewer germ cell-like cells *in vivo* with distinct defects in gene expression. The results indicate that xeno-transplantation of hiPSCs directs germ cell differentiation in a manner dependent on donor genetic background (Ramathal *et al.*, 2014). Theoretically, iPSCs from male infertility patients with genetic defects could be genetically corrected, differentiated into PGCLCs or spermatogonia *in vitro*, and then transplanted back into the patient’s seminiferous tubules for therapeutic purpose. Moreover, xeno-transplantation of iPSCs may also serve as tools for genetic research of human germ cell development *in vivo* (Fig. 1).

Recently, several groups utilized the embryoid body differentiation strategy to achieve *in vitro* induction of human PGCLCs from iPSCs in response to cytokines resembling those released by early extra-embryonic tissues and involved in critical germ cell fate regulation pathways. Irie *et al*. (2015) reported that human PGCLCs specification could be induced efficiently and directly in hiPSCs that were maintained in the 4i medium. Furthermore, Sasaki *et al*. (2015) showed that hiPSCs in primed state could differentiate into incipient mesoderm-like cells by stimulation with Activin A and a WNT signaling agonist (CHIR99021), and then generated PGCLCs in response to growth factors, robustly yielding B-lymphocyte-induced maturation protein 1 (BLIMP1, officially known as PRDM1) and TFAP2C activated cells. Importantly, the authors also demonstrated that transcriptomes of the obtained PGCLCs were similar to PGCs isolated from non-human primates. With different cytokine combinations, Sugawa *et al*. (2015) also described a defined and stepwise differentiation system for inducing pre-migratory PGCLCs from hiPSCs. Moreover, the PGCLCs they generated showed epigenetic reprogramming that was globally similar to PGCs *in vivo*.

Taken together, these studies indicate that human male germ cells can be derived from hiPSCs, although most of the differentiated cells remained at the early stages, like PGCLCs. Therefore, development of *in vitro* conditions that enable robust differentiation of human PGCLCs towards later stages will be necessary.

### Prospectives of hiPSCs in male infertility

#### Reproductive medicine applications

The process of generating male gametes from patient-specific iPSCs could provide better *in vitro* disease models for male infertility. Comparison of patient-derived hiPSCs with normal hiPSCs for their germ line differentiation abilities may help identify abnormalities and decipher the molecular mechanisms of idiopathic male infertility involved in differentiation and maturation of human gametes. Furthermore, differentiation of male germ cells from hiPSCs would be an invaluable tool to explore the specification of human germ line development, including transcriptional networks, signaling pathways and epigenetic reprogramming. In contrast to studies in mice, studies of human germ line development are limited mainly due to inaccessibility of germ cells during early embryo development and lack of suitable experimental systems (Durruthy-Durruthy *et al.*, 2014). So it is necessary to reconstitute human germ line development *in vitro*, which could possibly be achieved by differentiation of hiPSCs.

In addition, the establishment of functional male gametes from autologous iPSCs could benefit azoospermia patients who lack mature sperm in the testes. However, the limited publications about haploid spermatid indicate that the derivation of functional gametes from hiPSCs is still an immature technology and insufficient for clinical therapies (Nikolic *et al.*, 2016). Moreover, germ cells go through dramatic epigenetic reprogramming during development (Tang *et al.*, 2016). The evaluation of epigenetic status during germ cell differentiation from hiPSCs is informative and is not fully evaluated in most published studies.

#### Reconstitution of spermatogenesis niche

The differentiation of male germ cells *in vivo* depends on a niche composed of spermatogenic and somatic cells. It is important to reconstitute the spermatogenic niche to support differentiation of iPSCs into functional germ cells. However, testing whether human PGCLCs can form spermatozoa *in vivo* is not feasible for ethical reasons and tumorigenicity after transplantation into human testis. Instead, xeno-transplantation of hiPSCs and PGCLCs into murine seminiferous tubules could provide a somatic environment to promote human germ line formation *in vivo* (Durruthy-Durruthy *et al.*, 2014; Ramathal *et al.*, 2014).

Additionally, it was demonstrated that co-culture with neonatal testicular somatic cells and sequential exposure to morphogens and sex hormones could promote mESC-derived PGCLCs to recapitulate complete male gametogenesis *in vitro* (Zhou *et al.*, 2016). Given the limited availability of fetal and neonatal human testicular biopsies, few studies have used the co-culture method to examine human germ cell development. Recently, a 3D testicular cell culture has been established to mimic the testis environment *in vivo* (Yokonishi *et al.*, 2013). The single testicular cells of neonatal mice formed aggregates in suspension culture and then were transferred to the surface of agarose gel with a gas-liquid interphase culture method. A tubular architecture gradually developed during the following 2 weeks. Furthermore, they also mixed spermatogonial stem cells (SSCs) with the testicular cell suspension and found the incorporation of SSCs in the reconstructed tubules. 3D cultures of hiPSCs and PGCLCs with testicular cells using biomaterials and bioactive factors will offer a new avenue for *in vitro* germ cell differentiation (Galdon *et al.*, 2016; Sadri-Ardekani and Atala, 2015).

#### Genome editing in hiPSCs

Male infertility is a multi-factorial disease with at least 15% of cases attributed to genetic disorders (Krausz *et al.*, 2015). Mutations carried by germ cells can lead to disorders in the offspring, and natural selection prevents the transmission of mutations by causing infertility, but this protective mechanism may be overcome by ART (Ferlin *et al.*, 2007). By using optimized gametes for ART, one could obtain healthy offspring without genetic disorders. *In vitro* derivation of germ cells from patient-specific iPSCs combined with genetic defect correction may provide valuable insights into the targeted treatments for infertility.

The rapid development of novel genome editing technologies, especially with the Clustered Regularly Interspaced Short Palindromic Repeats/CRISPR-associated protein-9 nuclease (CRISPR/Cas9) system, could realize the full potential of hiPSCs in both basic research and therapies for male infertility. In the past few years, there has been a spike of interest in genome editing in hESCs and hiPSCs, due to their potential in modeling and correcting a variety of genetic diseases (Cherry and Daley, 2013; Soldner and Jaenisch, 2012). For male infertility caused by genetic anomalies, genome editing technologies could be used to target and modify the gene of interest in patient-specific iPSCs before they are differentiated and introduced to patients (Li *et al.*, 2014a,b). Other than cell and gene therapy, genome editing technologies can also facilitate basic research by generating knock-in reporters under the control of regulatory elements of germ cells to better visualize key stages during germ line development from iPSCs *in vitro* (Irie *et al.*, 2015; Sasaki *et al.*, 2015).

Genome editing technologies will continue to develop for clinical applications, offering hope to infertile male patients with genetic disorders, but also raising ethical arguments, long-term safety issues and even unpredictable impacts on humans. In this regard, future use of genome editing in the clinic requires extra long-term evaluation of the safety of cells that have undergone genome editing.

#### Human iPSCs-derived extracellular vesicles and male infertility

Male infertility is a common iatrogenic effect of clinical treatment for cancer, including radiotherapy and high-dose chemotherapy, which can severely damage the male gonad leading to spermatogenic failures (Jahnukainen and Stukenborg, 2012; Martin-du Pan and Campana, 1993). Emergence of iPSC-derived germ cells presents a valuable opportunity to replenish autologous germ cells for male infertility patients. Nevertheless, the therapeutic application of iPSCs and their differentiated derivates are limited by their tumorigenicity (Lee *et al.*, 2013). Extracellular vesicles (EVs) are membrane-bound vesicles carrying regulatory molecules, such as microRNAs, proteins and lipids. They may mediate intercellular communications, contributing to cell proliferation and differentiation (Machtinger *et al.*, 2016). It has been demonstrated that exosomes and microvesicles secreted by iPSCs are very effective transmitters of cytoprotective signals to cardiomyocytes in the setting of myocardial ischemia/reperfusion (Wang *et al.*, 2015). Spermatogenesis is a complex process highly dependent on intercellular communications among germ cells, Sertoli cells and Leydig cells. These intercellular communications could be closely related to the biological functions of EVs. So it is hypothesized that iPSC-derived exosomes and microvesicles could transmit cytoprotective signals to the injured spermatogenic microenvironment caused by anticancer treatment and promote the recovery of testicular spermatogenic function without the risk of tumorigenicity.

## Conclusion

Although controversial, hiPSCs have tremendous potential for biological and therapeutic applications for male infertility. Recent advances in generation of male germ cells from miPSCs not only hold great promise for the establishment of *in vitro* human spermatogenesis models, but also provide insights into the mechanism of hPGC specification and human spermatogenesis regulation. Based on these advances, it is conceivable that hiPSCs will have more therapeutic implications for male infertility in combination with genome editing technology and EVs research in the near future. However, the molecular mechanisms underlying human male germ cell development are still poorly understood. More comprehensive understanding of human germ cell development would be of great value for the application of hiPSCs in reproductive medicine and basic research.

## Authors’ roles

F.F. designed the review, performed the literature research and drafted the article. Z.L.L. designed the review, performed revisions and critically discussed the complete article. Q.Z. performed revisions and critically discussed the complete article. C.L.X. and H.G.L. designed the review, supervised and revised it critically for important intellectual content.

## Funding

National Science & Technology Pillar Program during the 12th 5-Year Plan Period, China (No. 2012BAI32B03) and National Natural Science Foundation of China (No. 81370755).

## Conflict of interest

The authors report no financial or other conflict of interest relevant to the subject of this article.
